# Genome-wide identification and co-expression network analysis provide insights into the roles of auxin response factor gene family in chickpea

**DOI:** 10.1038/s41598-017-11327-5

**Published:** 2017-09-07

**Authors:** Vikash K. Singh, Mohan Singh Rajkumar, Rohini Garg, Mukesh Jain

**Affiliations:** 1 National Institute of Plant Genome Research (NIPGR), New Delhi, India; 2 School of Natural Sciences, Department of Life Sciences, Shiv Nadar University (SNU), Dadri, Uttar Pradesh India; 3 School of Computational and Integrative Sciences (SC&IS), Jawaharlal Nehru University (JNU), New Delhi, India

## Abstract

Auxin response factors (ARFs) are the transcription factors that regulate auxin responses in various aspects of plant growth and development. Although genome-wide analysis of ARF gene family has been done in some species, no information is available regarding ARF genes in chickpea. In this study, we identified 28 ARF genes (CaARF) in the chickpea genome. Phylogenetic analysis revealed that CaARFs can be divided into four different groups. Duplication analysis revealed that 50% of *CaARF* genes arose from duplication events. We analyzed expression pattern of *CaARFs* in various developmental stages. *CaARF16*.*3*, *CaARF17*.*1* and *CaARF17*.*2* showed highest expression at initial stages of flower bud development, while *CaARF6*.*2* had higher expression at later stages of flower development. Further, *CaARF4*.*2*, *CaARF9*.*2*, *CaARF16*.*2* and *CaARF7*.*1* exhibited differential expression under different abiotic stress conditions, suggesting their role in abiotic stress responses. Co-expression network analysis among *CaARF*, *CaIAA* and *CaGH3* genes enabled us to recognize components involved in the regulatory network associated with CaARFs. Further, we identified microRNAs that target *CaARFs* and *TAS3* locus that trigger production of trans-acting siRNAs targeting *CaARFs*. The analyses presented here provide comprehensive information on ARF family members and will help in elucidating their exact function in chickpea.

## Introduction

Auxin or indole-3-acetic acid (IAA), a crucial phytohormone, plays a vital function in regulation of numerous aspects of growth and development in plants. Many studies have shown role of auxin in regulation of several biological processes, such as apical dominance, embryo patterning, formation of lateral root, shoot elongation, tropic responses and vascular differentiation^1–4^. Auxin response factors (ARFs) are important transcription factors that can activate or repress the expression of early/primary auxin response genes [Auxin/Indole-3-acetic acid (Aux/IAA), Small Auxin Up RNA (SAUR) and Gretchen Hagen 3 (GH3)] via binding with auxin response elements (AuxREs, TGTCTC) or some variation of these elements (TGTCCC or TGTCAC) in their promoters^5–7^. Recently, microarray experiments indicated that AtARF1 and AtARF5 prefer to bind TGTCGG elements as compared to AuxRE TGTCTC^8^. TGTCGG appeared to be the preferred DNA binding motif of ARF2 and ARF5 in a “cistrome” analysis as well^9^.

ARF proteins contain three domains, i.e. a N-terminal B3-like DNA binding domain (DBD), C-terminal PB1 (Phox and Bem1) domain contained within a region that was previously called motif III/IV, and a middle region (MR), which is responsible for gene activation/repression^8, 10–15^.

ARF gene family has been analyzed in many plants, such as Arabidopsis, rice, Medicago and tomato^2, 10, 16–18^. Function of many ARF proteins have also been studied in plants. For instance, *arf1* and *arf2* loss-of-function mutants in Arabidopsis exhibited abnormal abscission of floral organs and senescence in leaf tissues^19^, while *arf3/ett* illustrated abnormal floral meristem patterning and gynoecium development^20^. In addition, *arf5* mutant revealed abnormality in formation of embryo axis and vascular strands^21^. Furthermore, response of hypocotyl to blue light and auxin stimulus was found to be affected by mutation in *AtARF7* ^22^. However, double mutants of *arf6/arf8* were found to have infertile closed buds with short petals and stamen filaments besides bearing undehisced anthers^23^. Auxin-dependent lateral root development was found to be hampered in double mutants of *arf7/arf*19^24^. The antisense *OsARF1* rice transgenic lines exhibited altered organ size, curled leaves, poor vigor and reduced growth, indicating its role in development of somatic and reproductive tissues^25^. In addition, OsARF16 was found to be involved in iron and phosphate starvation responses in rice^26, 27^.

Chickpea (*Cicer arietinum* L.) is a model legume crop rich in dietary proteins and fibers for humans and animals. Since ARFs orchestrate various developmental processes in plants and their genetic manipulation holds potential for generating better yielding crops^17, 28–30^, it is important to study this gene family in chickpea. In this study, we identified members of ARF gene family in the chickpea genome. Duplication analysis revealed expansion of ARF gene family in chickpea via segmental duplication. Comprehensive gene expression profiling revealed significant differential expression of several ARF genes across diverse tissue types, indicating functional divergence of this gene family. A co-expression network of chickpea ARF, IAA and GH3 genes was generated and candidate genes involved in developmental processes were identified. These analyses along with prediction of ARFs as targets of miRNA and tasiRNA will help in understanding auxin response in chickpea.

## Results and Discussion

### Discovery of ARF gene family in chickpea

To define members of ARF gene family in chickpea, protein sequences of 23 known ARFs in Arabidopsis (AtARFs) were used to identify their homologs in chickpea genome using BLAST searches. In addition, chickpea proteome was used to perform HMM (Hidden Markov Model) profile searches. The list of putative members of ARF gene family obtained from the above two approaches were merged to generate a unique gene list. To confirm the presence of ARF domain, protein sequences of putative ARF gene family members of chickpea were analyzed in Pfam and SMART databases. In total, 28 protein sequences were confirmed as ARFs in chickpea (CaARFs). Naming of chickpea ARF genes was done in accordance to their evolutionary relationship with Arabidopsis ARFs (Table S1).

The size of CaARF proteins ranged from 279 (CaARF5.2) to 1089 (CaARF19) amino acids. Their isoelectric points varied from 5.55 (CaARF5.1) to 8.56 (CaARF10.2), indicating that different CaARF proteins may function in different microenvironments.

### Determination of gene structure and evolutionary relationship

**Figure 1:**
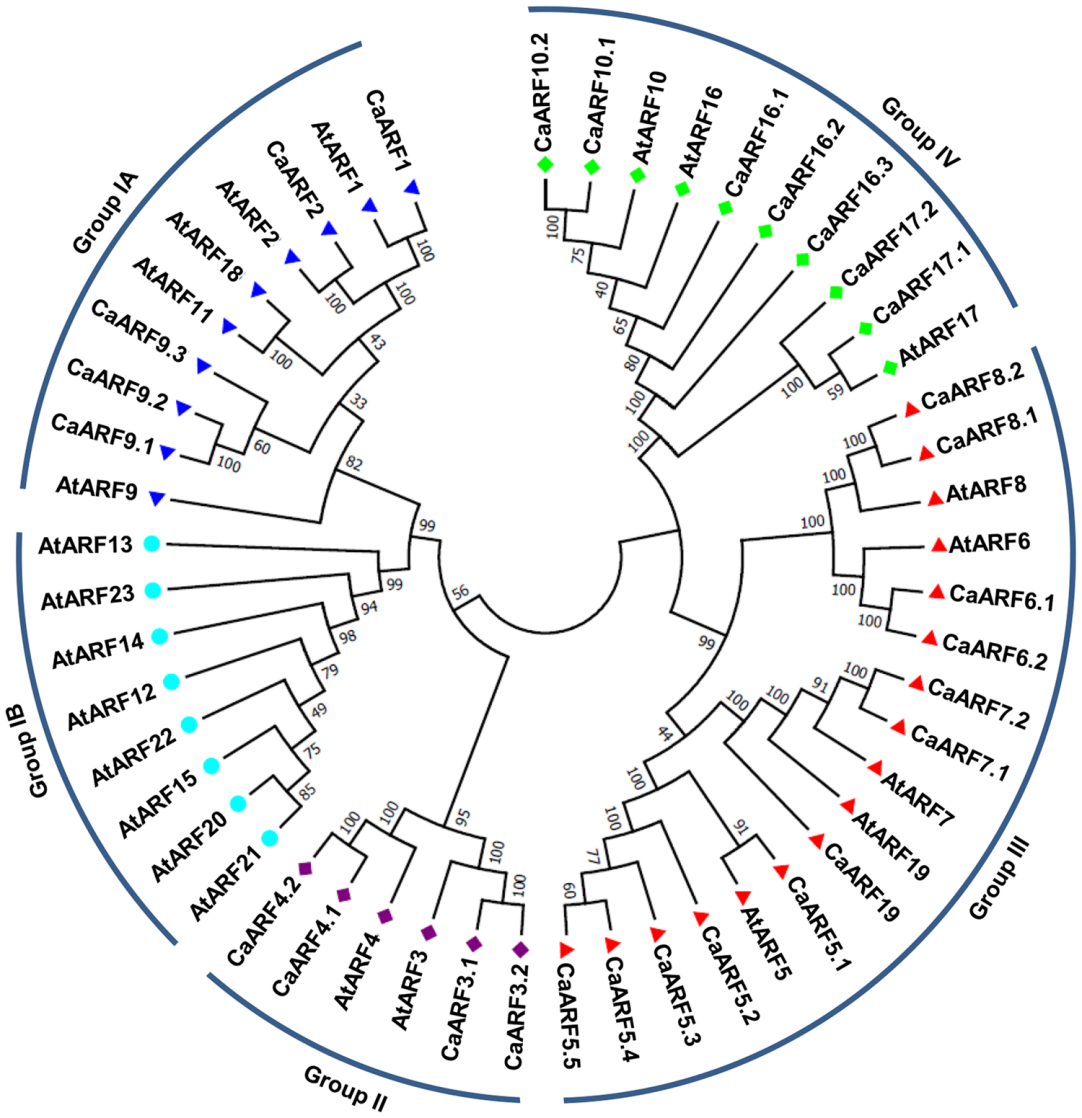
Phylogenetic relationship among ARF proteins from chickpea and *Arabidopsis*. The deduced full-length amino acid sequences of chickpea (CaARF) and Arabidopsis (AtARF) genes were aligned by MUSCLE and the phylogenetic tree was constructed by MEGA (v7.0) using the neighbour joining (NJ) method. The numbers on the nodes represent bootstrap values from 100 replicates. Grouping of ARFs has been indicated in different colors.

**Figure 2:**
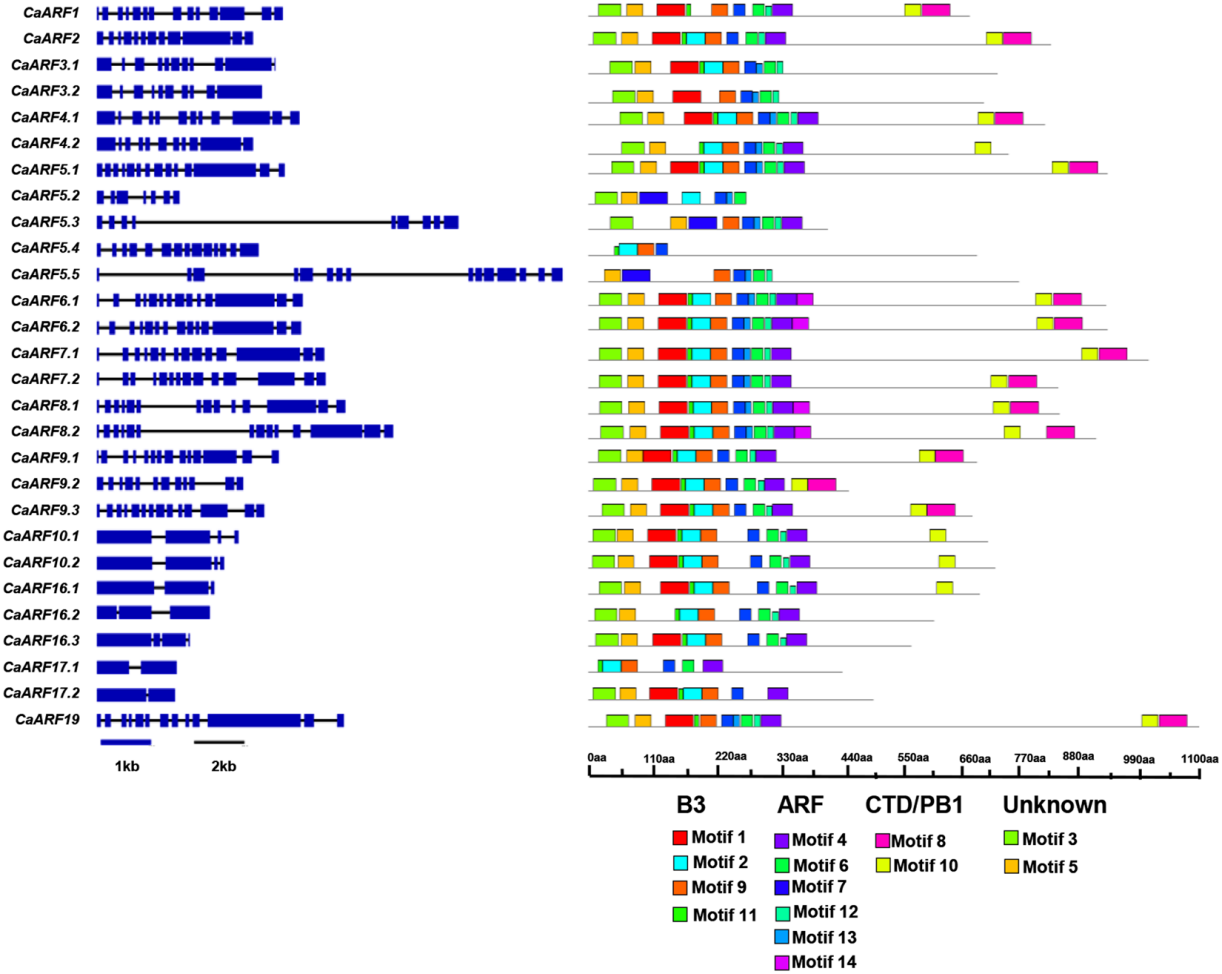
Gene structure and motif organization of ARF genes in chickpea. *Left* panel illustrates the exon–intron organization of ARF genes. Exons and introns are represented by boxes (blue) and lines (black), respectively. *Right* panel shows motif organization in ARF proteins. Motifs 1, 2, 9 and 11 represent B3-domain; Motifs 4, 6, 7, 12, 13 and 14 represent ARF-domain; Motifs 8 and 10 represent PB1-domain; Motifs 3 and 5 are unknown novel motifs.

An unrooted phylogenetic tree was constructed using protein sequence alignment of ARF gene family members from Arabidopsis (23) and chickpea (28) for examining evolutionary relationship among them (Fig. 1). Phylogenetic analysis grouped ARF proteins into four major classes, I (A and B), II, III and IV (Fig. 1). Group IA consisted of five CaARF proteins and five AtARFs. Notably, group IB lacked any CaARF protein and was comprised of only eight AtARFs (Fig. 1). Six ARFs were clustered in group II (four from chickpea and two from Arabidopsis). Group III harbored 17 members (12 CaARFs and five AtARFs), whereas clustering of 10 members (seven CaARFs and three AtARFs) was observed in group IV. Most of CaARF paralog pairs identified from phylogenetic tree analysis were present on duplicated chromosomal blocks (Fig. 1). Similarity of sequences between paralog pairs was quite high, indicating that these genes might be involved in governing similar functions. Furthermore, we identified at least 14 conserved motifs in the CaARF protein sequences using MEME (Fig. 2, Table S2a,b). Among these, four motifs (1, 2, 9 and 11) were found to be associated with N-terminal B3-domain, six motifs (4, 6, 7, 12, 13 and 14) associated with middle ARF domain, while motifs 8 and 10 were associated with C-terminal PB1 domain. In addition, we identified two novel motifs (motifs 3 and 5) located at the N-terminus of most of CaARF protein sequences (Fig. 2). Their function needs to be recognized. Corroborating our predictions, members grouped together in the phylogenetic tree revealed similar motif organization with few exceptions (for example, motifs 3 and 5 were absent in CaARF5.2, 5.3, 5.4 and 5.5 as compared to CaARF5.1), signifying their functional coherence (Fig. 2). Further, we analyzed exon-intron organization of all CaARFs genes. Although the number of exons varied from 2 to 17 (Fig. 2), members of the same group represented similar exon-intron structure.

### Genomic location and gene duplication events in CaARF gene family

**Figure 3:**
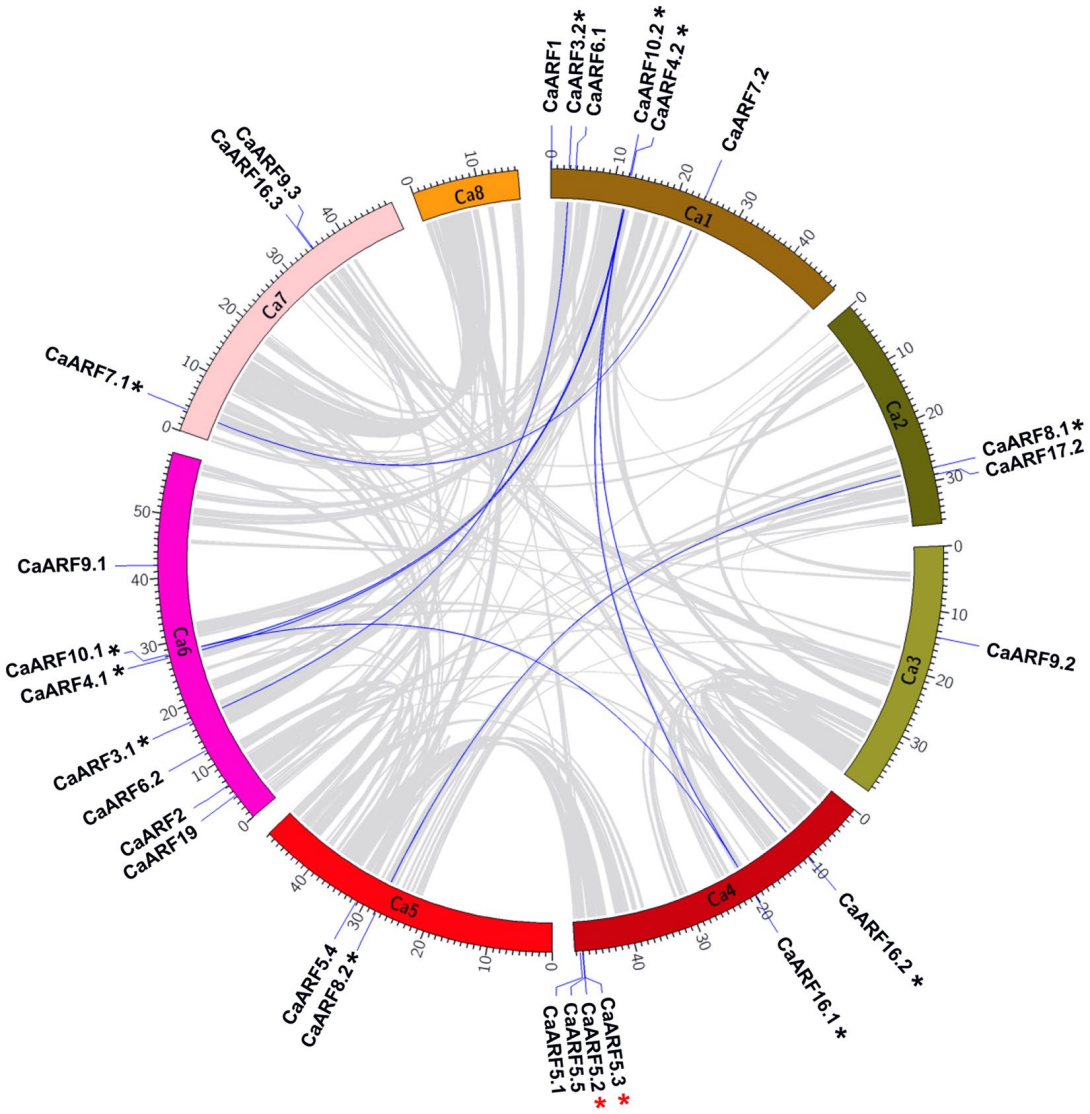
Mapping of duplicated *CaARF* genes on chickpea chromosomes. Grey ribbons indicate collinear relationship among the blocks in whole genome and blue ribbons show *CaARF* paralogs. The chickpea chromosomes are arranged in a colored arc and the size of each arc corresponds to the size of respective chromosome (Mb). Genes with black asterisk represent segmentally duplicated genes and red asterisks highlight the tandemly duplicated genes.

Physical position of 27 CaARF genes was found on seven chromosomes of chickpea (except chromosome 8), while one CaARF gene (*CaARF17*.*1*) was present on a scaffold (Fig. 3). The number of ARF genes on different chromosomes ranged from one to seven (Fig. 3). One ARF gene was located on chromosome 3 (*CaARF9*.*2*), two genes on chromosome 2 (*CaARF8*.*1* and *17*.*2*) and chromosome 5 (*CaARF5*.*4* and *8*.*2*) each, three genes on chromosome 7 (*CaARF7*.*1*, *9*.*3* and *16*.*3*), six genes on chromosome 1 (*CaARF1*, *3*.*2*, *6*.*1*, *10*.*2*, *4*.*2* and *7*.*2*) and chromosome 4 (*CaARF16*.*2*, *16*.*1*, *5*.*3*, *5*.*2*, *5*.*5* and *5*.*1*) each, and seven ARF genes were located on chromosome 6 (*CaARF2*, *3*.*1*, *4*.*1*, *6*.*2*, *9*.*1*, *10*.*1* and *19*) (Fig. 3).

Duplication analysis revealed expansion of ARF gene family in chickpea. Among the 28 ARF genes, 14 (50%) arose from duplication events, including twelve genes (*CaARF3*.*1/3*.*2*, *CaARF10*.*1/10*.*2/16*.*1/16*.*2*, *CaARF4*.*1/4*.*2*, *CaARF7*.*1/7*.2 and *CaARF8*.*1/8*.2) representing segmental duplications (42.8%) and two genes (*CaARF5*.*2/5*.*3*) representing tandem duplications (7.1%) (Fig. 3). In Arabidopsis, 14 (60.8%) out of 23 ARFs arose from duplication events, representing seven segmentally (30.4%) and seven tandemly (30.4%) duplicated genes^31^. All seven tandemly duplicated ARF genes (30.4%) of Arabidopsis belonged to group IB^31^, which is absent in chickpea (this study) and other plants^2, 17, 18, 28–30^. In phylogenetic tree, all the duplicated *CaARF* gene pairs were found to be clustered together (Fig. 3). Furthermore, higher sequence similarity between duplicated gene pairs suggested that they are likely to participate in regulation of similar biological processes. For example, *CaARF5*.*2* and *CaARF5*.*3* representing tandem duplication had 48.5% similarity, while *CaARF8*.*1* and *CaARF8*.*2* representing segmental duplication showed 84.2% similarity at the protein sequence level. In group III, one pair of tandemly duplicated genes (*CaARF5*.*2*/*5*.*3*) and one pair of segmentally duplicated genes (*CaARF8*.*1*/*8*.*2*) were present. The members grouped within subfamilies II and IV, represented segmental duplications (Figs 1 and 3). Thus, the expansion of chickpea ARF gene family may be attributed mainly due to segmental duplications, which is consistent with results found in other plants^18, 30, 32^.

Further, the approximate date of CaARF gene duplication events was estimated via determining *Ks* and *Ka* values along with *Ka*/*Ks* ratio (Table S3). *Ka*/*Ks* ratio of <1 suggests purifying selection, while *Ka*/*Ks* ratio >1 indicates possibility of positive selection^33, 34^. Further, *Ka*/*Ks* ratio gives insight into the selection pressure on amino acid substitutions. Usually, selection pressure suggests selective advantage for the altered amino acid residues in a protein and is essential for understanding functional residues and shift in protein function^35^. The tentative date for segmental gene duplications ranged from 46.7 Mya (*Ks = *0.56) for the paralogous pair *CaARF8*.*1/8*.*2* to 117.7 Mya (*Ks* = 1.42) for *CaARF10*.*2*/*16*.*1*. *Ka*/*Ks* ratio for all the *CaARF* paralogous pairs was less than 1, suggesting that they are under strong purifying selection. Among these, *Ka*/*Ks* ratio of three paralogous pairs (*CaARF10*.*2*/*16*.*2*, *CaARF3*.*1*/*3*.*2* and *CaARF4*.*1*/*4*.*2*) was found to be more than 0.3, suggesting possibility of significant functional divergence after duplication. Five paralogous pairs (*CaARF10*.*2*/*16*.*1*, *CaARF10*.*1*/*10*.*2*, *CaARF7*.*1*/*7*.*2*, *CaARF8*.*1*/*8*.*2* and *CaARF10*.*1*/*16*.*1*) showed *Ka*/*Ks* ratio less than 0.3, suggesting their functional conservation.

### Differential expression and promoter analysis of CaARF genes

**Figure 4:**
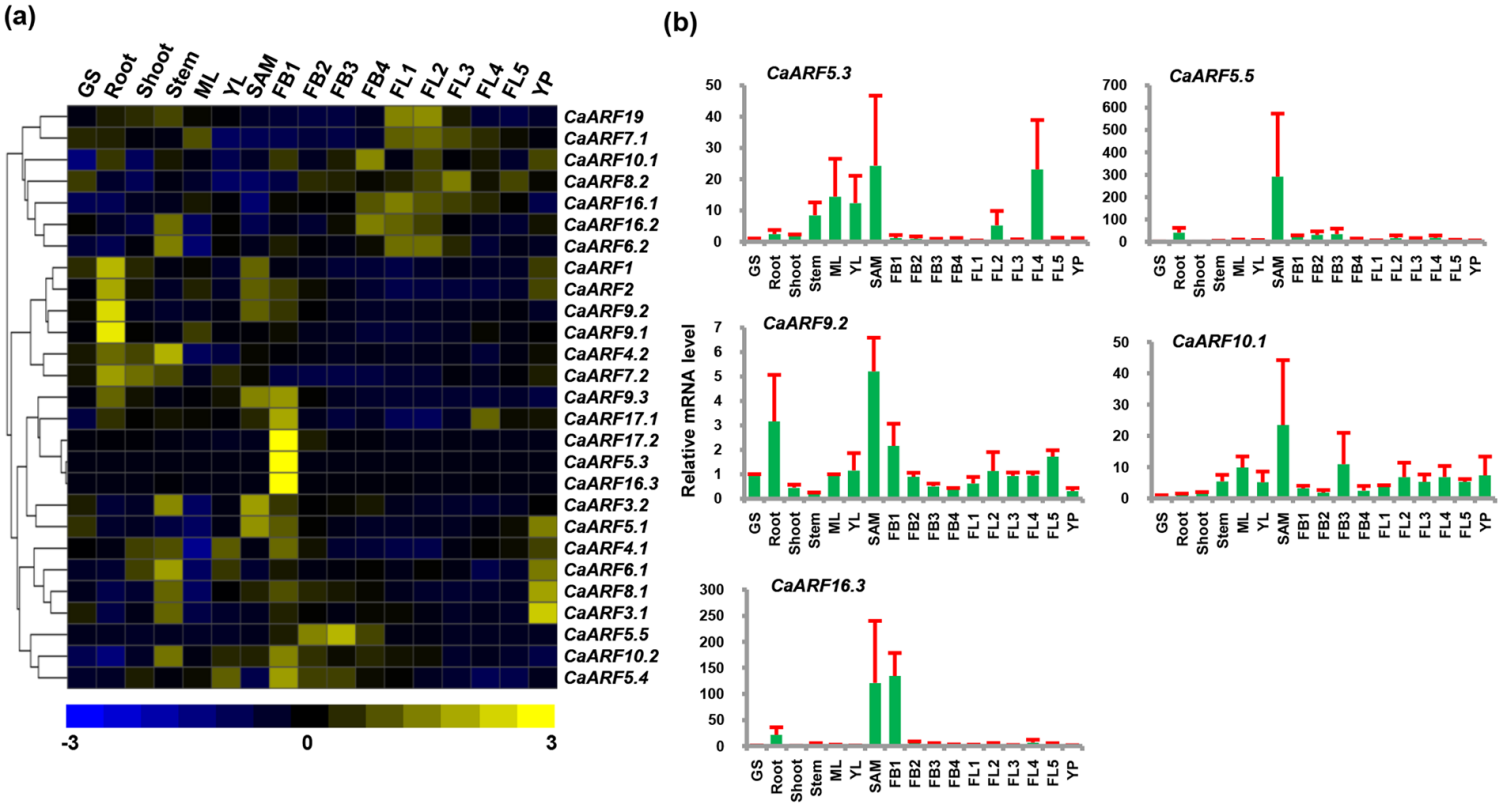
Expression profiles of *CaARF* genes in different tissue/organs. (**a**) Heatmap showing the normalized RNA-seq expression (FPKM) transformed into Z-score. Hierarchical clustering was conducted in R using the pheatmap package with dissimilarity metrics based on Euclidean distance with complete linkage rule. Color key at the bottom represents row wise Z-score. (**b**) RT–qPCR analysis of *CaARF* genes in various tissue/stages of development. Expression of germinating seedling (GS) was taken as a reference to determine relative mRNA level in all other tissues for each gene. Error bars indicate standard error (SE) of mean. *GS*, germinating seedling; *ML*, mature leaf; *YL*, young leaf; *SAM*, shoot apical meristem; *FB1*–*4*, stages of flower bud; *FL1–5*, stages of flower; *YP*, young pod.

To investigate putative roles of *CaARF* genes, expression pattern of all *CaARFs* was studied in diverse organs/tissues representing vegetative and reproductive stages using RNA-seq data^36, 37^. Many of *CaARFs* exhibited a distinct tissue-specific expression pattern (Fig. 4). For example, *CaARF1*, *2*, *9*.*1* and *9*.*2*, belonging to group IA, exhibited higher transcript accumulation in root as compared to any other tissue, indicating that they may function in root development (Fig. 4a, Table S4). Interestingly, *CaARF1* and *2* are putative orthologs of Arabidopsis, *AtARF1* and *2* (group IA), respectively, that were also found to have higher expression in primary root tips^38^. *CaARF16*.*3*, *17*.*1* and *17*.*2*, which belong to group IV showed higher expression specifically in FB1 stage of flower development (Fig. 4a, Table S4). *AtARF8* is known to regulate flower maturation and fertilization in Arabidopsis^23, 39^. Interestingly, its putative orthologs in chickpea, *CaARF8*.*1* and *CaARF8*.*2*, showed higher expression at later stages of flower development. (Fig. 4a, Table S4). *CaARF8*.*1* and *CaARF8*.*2* were found to be segmentally duplicated, suggesting that their subfunctionalization after duplication might have resulted into partitioning of their function in different aspects of reproductive development in chickpea. *AtARF6* have been reported to be involved in flower maturation along with *AtARF8* ^23, 39^. Its ortholog, *CaARF6*.*2*, also exhibited higher expression at later stages of flower development (Fig. 4a, Table S4). *AtARF3/ETTIN* is known to integrate function of *AGAMOUS* (*AG*) and *APETALA2* (*AP2*) in floral meristem determinacy^40^. Interestingly, its ortholog *CaARF3*.*2* exhibited greater transcript accumulation in shoot apical meristem (SAM, Fig. 4). In addition, AtARF3/ETTIN interacts with KANADI proteins to form a functional complex required for leaf polarity^41^. Furthermore, AtARF5/MONOPTEROS has been reported to regulate flower formation^21^. Its putative orthologs in chickpea, *CaARF5*.*1*, *5*.*3*, *5*.*4* and *5*.*5*, showed enhanced expression at flower bud stages (Fig. 4a, Table S4). *CaARF5*.*3* and *CaARF5*.*5* were found to be expressed specifically but at very low levels at FB1 (FPKM = 0.02) and FB3 (FPKM = 1.62) stages, respectively. Likewise, *CaARF6*.*2*, *7*.*1*, *8*.*2*, *10*.*1*, *16*.*1*, *16*.*2*, and 19, which clustered together (Fig. 4a, Table S4), showed increased transcript accumulation at different stages of flower development, suggesting their possible role during flower development. The expression profile of at least five *CaARF* genes (*CaARF5*.*3*, *5*.*5*, *9*.*2*, *10*.*1* and *16*.*3*) was analyzed through RT-qPCR to validate the RNA-seq results. The expression patterns obtained via RT-qPCR were well correlated with that of RNA-seq (Fig. 4a,b).

The temporal and spatial gene expression is influenced by the presence of different *cis*-regulatory elements in the promoters, where transcription factors can bind. We analyzed *cis*-regulatory elements in the promoter sequences of *CaARF* genes using PlantPAN 2.0 database^42^. Most of *CaARF* promoter sequences revealed the presence of canonical TATABOX 1 and CAATBOX 1 (Table S5). In addition, a variety of light signalling related elements, such as GBOXLERBCS, GT1GMSCAM4 and BOXIIPCCHS were found to be present in *CaARF* promoters (Table S5) indicating their possible role in crosstalk between auxin and light signalling pathway^43^. Further, many hormone-responsive elements, namely, ABREATRD22, TCA1MOTIF, T/GBOXATPIN2, GAREAT, WBOXATNPR1, GCCCORE, C2GMAUX28, D1GMAUX28 and D3GMAUX28 were also detected in their promoters (Table S5), suggesting that *CaARFs* are regulated by auxin and other hormones.

### Differential expression of ARF genes under abiotic stress

**Figure 5:**
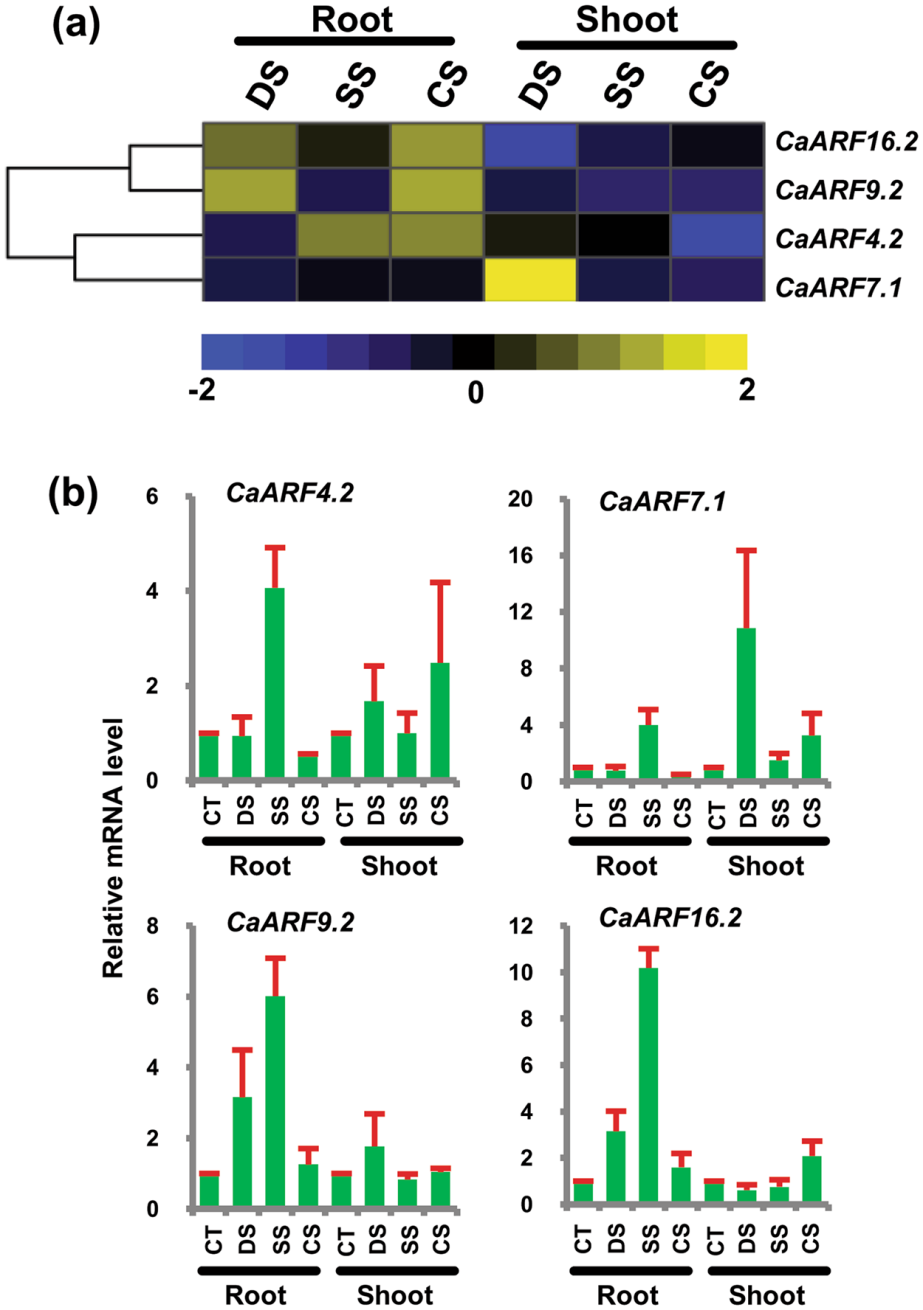
Expression profiles of *CaARF* genes under abiotic stress conditions. (**a**) Heatmap showing differential expression of *CaARFs* based on RNA-seq data. Color key at the bottom represents log_2_ fold change. (**b**) RT–qPCR analysis of *CaARF* genes under various stress treatments. Root and shoot control were taken as a reference to determine relative mRNA level under stress conditions. Error bars indicate standard error (SE) of mean. *DS*, desiccation; *SS*, salinity; *CS*, cold stress, *CT*, control.

Promoter sequences of CaARFs exhibited the presence of several abiotic stress response related elements, including dehydration-responsive element (DRECRTCOREAT), low temperature-responsive element (LTRE1HVBLT49), ABA-responsive element (ABREATCONSENSUS, ABREATRD22, ABRELATERD1) and C-repeat binding factor (CBFHV) (Table S5), indicating their role in abiotic stress responses as well. Therefore, to understand the role of CaARFs in abiotic stress responses, we analyzed the RNA-seq data for abiotic stress-treated chickpea tissues^44^. We detected four genes, including *CaARF4*.*2*, *CaARF7*.*1*, *CaARF9*.*2* and *CaARF16*.*2*, which exhibited significant differential expression (fold change ≥ 2 and p-value ≤ 0.05) under different abiotic stresses (Fig. 5a, Table S6). For instance, *CaARF4*.*2* was significantly up-regulated under salt and cold stresses in root (Fig. 5a, Table S6), suggesting its involvement in salinity and cold stress responses. *CaARF9*.*2* and *CaARF16*.*2* were significantly up-regulated during desiccation and cold stresses in root samples (Fig. 5a, Table S6). In shoot, *CaARF7*.*1* was significantly up-regulated under desiccation (Fig. 5), suggesting its role in desiccation stress response. The differential expression of these *CaARF* genes under abiotic stress was analyzed via RT-qPCR as well (Fig. 5b). Although the expression profile of the *CaARF* genes in most of the samples could be validated via RT-qPCR, some differences between RNA-seq and RT-qPCR results were also observed (Fig. 5a,b). For instance, the differential expression of *CaARF4*.*2*, *CaARF9*.*2* and *CaARF16*.*2* in roots under cold stress revealed by RNA-seq, was not observed via RT-qPCR. Likewise, higher expression of *CaARF9*.*2* was observed in roots under salinity stress via RT-qPCR, which was not obvious in the RNA-seq results. It would be interesting to validate the mechanistic roles of these CaARFs in abiotic stress responses in chickpea.

### Co-expression network of CaARF, CaIAA and CaGH3 genes

**Figure 6:**
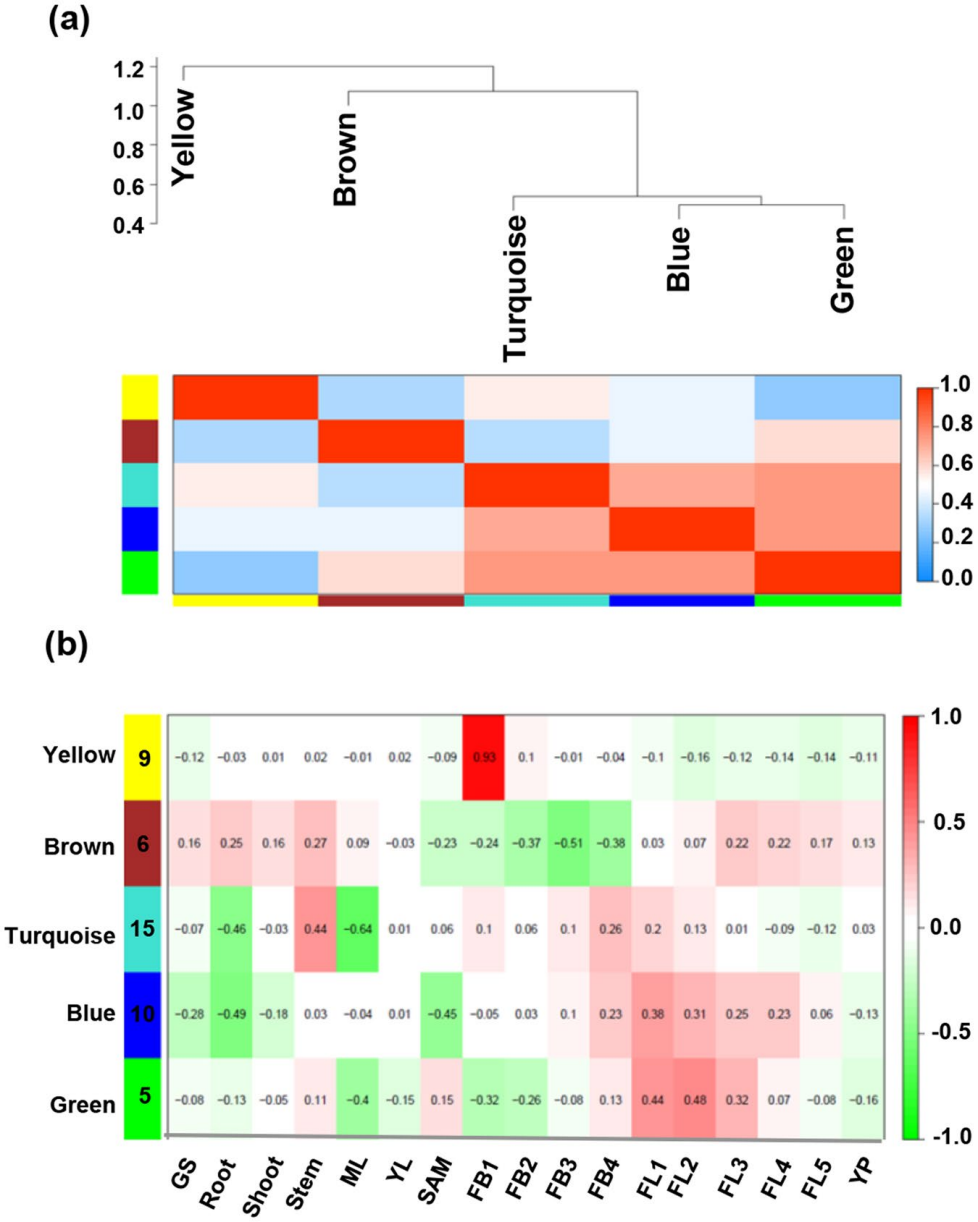
Co-expression network analysis of *CaARF*, *CaIAA* and *CaGH3* genes. (**a**) Cluster dendrogram showing correlation (0 to 1) between the modules. A high degree of correlation between modules is indicated by dark red color. (**b**) Module tissue correlation. Each row corresponds to a module. The number of genes in each module is indicated on the left. Each column corresponds to specific tissue labeled below. The color of each cell at row-column intersection indicates the correlation coefficient from −1 to 1. A high degree of correlation between a specific module and the tissue-type is indicated by dark red.

ARFs have been demonstrated to play a crucial role in plant development by interacting with other proteins especially with Aux/IAAs^13, 14^. ARF proteins activate or repress expression of auxin-responsive genes, which indicate the existence of a complex regulatory network between ARFs and other auxin-responsive genes. The middle region of ARF proteins is responsible for activation or repression depending on composition of amino acids^45^. The middle domain of repressor ARFs is enriched in proline, serine and threonine, whereas activators are enriched in glutamine^45^. Based on transactivation assay or middle domain prediction, five ARFs have been identified as activators (ARF5–ARF8 and ARF19) and rest as repressors except four truncated proteins (ARF3, ARF13, ARF17 and ARF23) in Arabidopsis^10^. Co-expression analysis is an important approach to explore potential function of genes. To understand the possible regulatory relationship of CaARFs with CaIAAs and CaGH3s, we performed co-expression network analysis using RNA-seq data^36, 37^ via Weighted Gene Co-expression Network Analysis (WGCNA)^46^ and detected highly interconnected modules of co-expressed genes (Fig. 6a,b). Normalized FPKM (fragments per kilobase of the transcript per million mapped reads) expression values in different tissues/organs^36, 37^ for *CaARF*, *CaIAA* and *CaGH3* genes were used as input for construction of co-expression network. Pearson correlation coefficient (PCC) with β = 8 (soft threshold power) was used to calculate an adjacency matrix. We detected five modules containing different sets of CaARF, CaIAA and CaGH3 genes based on their coexpression (Fig. 6a,b). Among all, turquoise module was the largest containing 15 genes, followed by blue (10 genes), brown (9 genes), yellow (6 genes) and green (5 genes) modules (Fig. 6b). Since all the modules have different set of genes and expression patterns, it is understandable that different cellular/developmental events might be the result of specific auxin response arising from differential transcriptional activity of ARF, Aux/IAA and GH3 family proteins.

**Figure 7:**
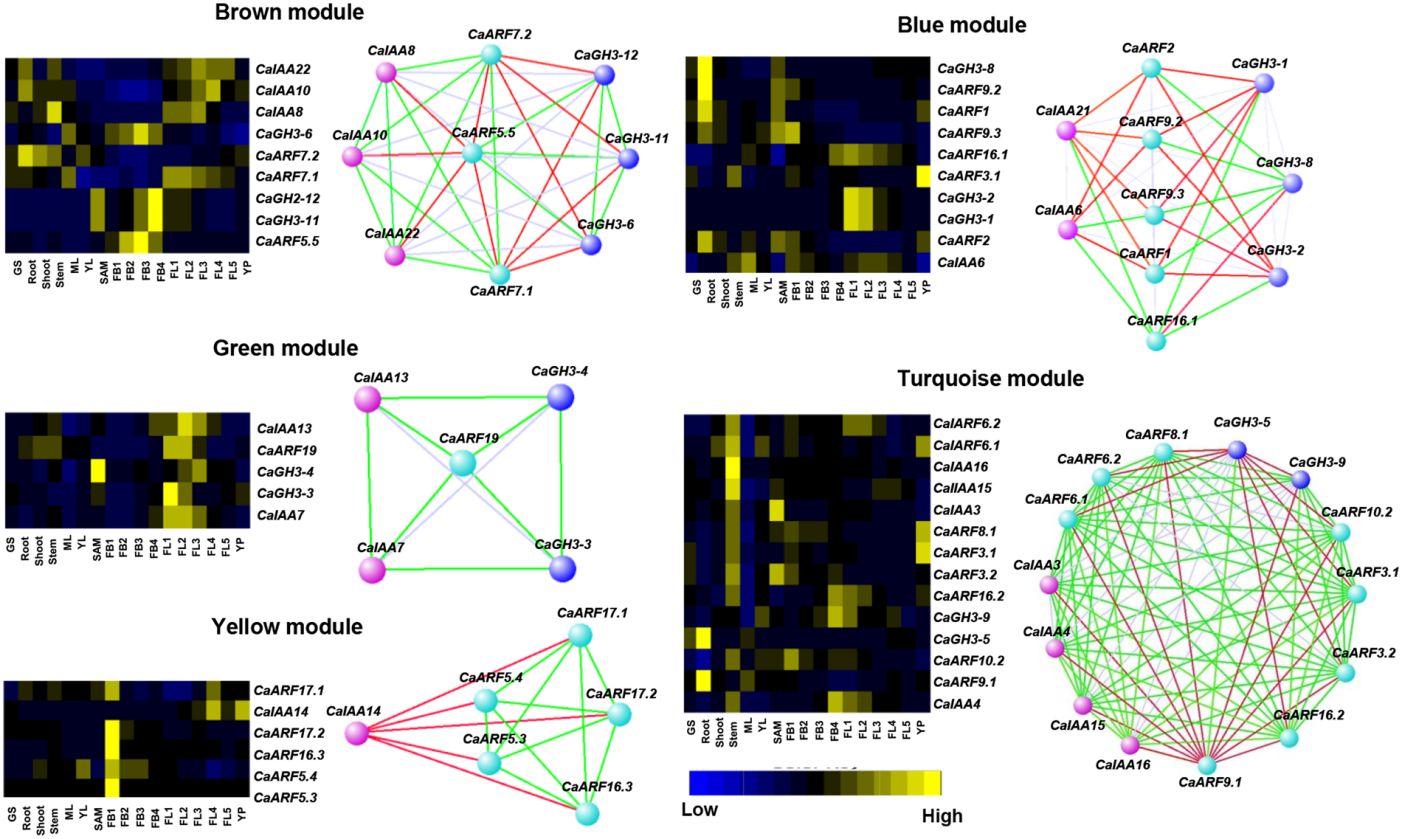
Co-expression modules and network of *CaARF*, *CaIAA and CaGH3* genes. *Left* panel shows heatmap depicting the expression of all genes included in each module. Color range from blue (lowest expression) to yellow (highest expression) represent expression level. *Right* panel shows co-expression network of genes in each module. Network is visualized in ViSANT. Spheres (nodes) represent genes, and green and red color lines (edges) represent positive and negative correlation, respectively.

It has also been shown that under high auxin concentration, ARFs form homodimers and activate auxin-responsive gene expression^45^. Several activator ARFs have been reported to interact with many Aux/IAA proteins in Arabidopsis and rice^45, 47, 48^. Co-expression analysis showed positive and negative correlation among the expression profiles of many of the *CaARF*, *CaIAA* and *CaGH3* members within modules. For instance, in green module, expression of *CaARF19* was found to be positively correlated with *CaIAA7*, *CaIAA13*, *CaGH3-3* and *CaGH3-4* (Fig. 7). Interaction between AtARF19 and several Aux/IAA proteins have been reported in Arabidopsis^47^. *AtARF8* was found to negatively regulate free IAA level by controlling expression of *GH3* genes in Arabidopsis^49^. The expression of *AtGH3-5*, *DFL1/AtGH3-6* and *AtGH3-17* was found to be decreased in *arf8* loss-of-function mutants and increased in *AtARF8* over expressing plants^49^, indicating that *AtARF8* can activate *GH3* gene expression. We hypothesize that CaARF19 can bind to the AuxRE elements residing in the promoter regions of *CaIAA7*, *CaIAA13*, *CaGH3-3* and *CaGH3-4* genes and induce their expression.

In brown module, *CaARF7*.*1* and *CaARF7*.*2* were positively correlated with *CaIAAs* and negatively correlated with other genes (*CaGH3-6*, 11, 12 and *CaARF5*.*5*), indicating that *CaIAAs* may form heterodimer with *CaARF7*.*1* or *CaARF7*.*2* for repressing the expression of downstream genes. In Arabidopsis also, several Aux/IAA proteins have been found interacting with AtARF7 too^47^. Similar correlations between *CaIAAs*, *CaARFs* and *CaGH3s* were observed for yellow and turquoise modules too (Fig. 7). Furthermore, *CaARF1*, *2*, *9*.*2*, *9*.*3* and *16*.*1*, were negatively correlated with *CaIAAs* (*CaIAA6* and 21) and *CaGH3s* (*CaGH3-1*, 2 and 8) in blue module, implying that higher expression of repressor *CaARFs* can inactivate downstream genes. It has been hypothesized that repressor ARFs can inhibit the activator ARF(s) via sequestering them by heterodimerization, with/without the binding of Aux/IAA protein(s) to the activator ARFs, or repressor ARFs might compete with activator ARFs for AuxRE binding sites, individually or as heterodimers, with or without the recruitment of other proteins such as TOPLESS^50^. A few CaARF proteins did not harbor one or the other of the three well-characterized functional domains (B3, ARF and CTD/PB1) as shown in Fig. 2. It will be interesting to elucidate the regulatory network of these CaARFs with different domain composition.

All the *CaARF* genes are yet to be functionally characterized. Via studying the expression patterns of CaARFs in diverse tissue types and construction of co-expression networks of *CaARFs*, we have created a framework for understanding the function of these uncharacterized genes in chickpea for future studies.

### Prediction of putative target *CaARF* genes of miRNAs and tasiRNA

MicroRNAs (miRNAs) are 21-nucleotide long RNAs that play an important role in regulation of gene expression in plants and animals^51–53^. In Arabidopsis, expression levels of *AtARF6* and *AtARF8* were found to be regulated by *miR167* ^54^, whereas *AtARF10*, *AtARF16* and *AtARF17* were targeted by *miR160* ^55^. To study potential regulation of CaARF genes by miRNAs, we predicted target CaARF genes of the known miRNAs in chickpea^56, 57^. We found that *CaARF6*.*1* and *CaARF6*.*2*, and *CaARF8*.*1* were targeted by *miR167* in chickpea also. In addition, *CaARF10*.*1* and *CaARF10*.*2*, *CaARF16*.*1*, *CaARF16*.*2* and *CaARF16*.*3*, and *CaARF17*.*1* and *CaARF17*.*2* were detected as targets of miR160. These results suggest that regulation of ARFs seems to be conserved in Arabidopsis and chickpea. Earlier reports also demonstrated conservation of *miR160* and *miR167*–target interactions throughout plant evolution^58–61^. In addition, we found that *CaARF3*.*2* was targeted by *Cat-miR395h*, whereas *CaARF5*.*2*, *CaARF5*.*3* and *CaARF5*.*5* were targeted by a novel miRNA, *Cat-NovmiR*4, identified in our previous study^56^. In Arabidopsis, 3′ cleavage products of *AtARF10*, *AtARF16* and *AtARF17* were detected in many tissues, indicating that *miRNA160* regulates these *ARFs* post-transcriptionally^62^. Further, Mallory *et al*.^62^ documented that miR160 directed post-transcriptional regulation of *AtARF17* is necessary for proper expression of certain *GH3*-like early auxin-responsive genes. The plants expressing a miRNA-resistant version of *AtARF17* showed enhanced *AtARF17* mRNA level and altered accumulation of auxin-responsive *GH3* mRNAs encoding for auxin-conjugating proteins. These expression changes were found to be correlated with many developmental defects, like defects in embryo, leaf symmetry and leaf shape, premature inflorescence development, reduction in petal size, abnormal stamen, sterility, and root growth defects^62^.

**Figure 8:**
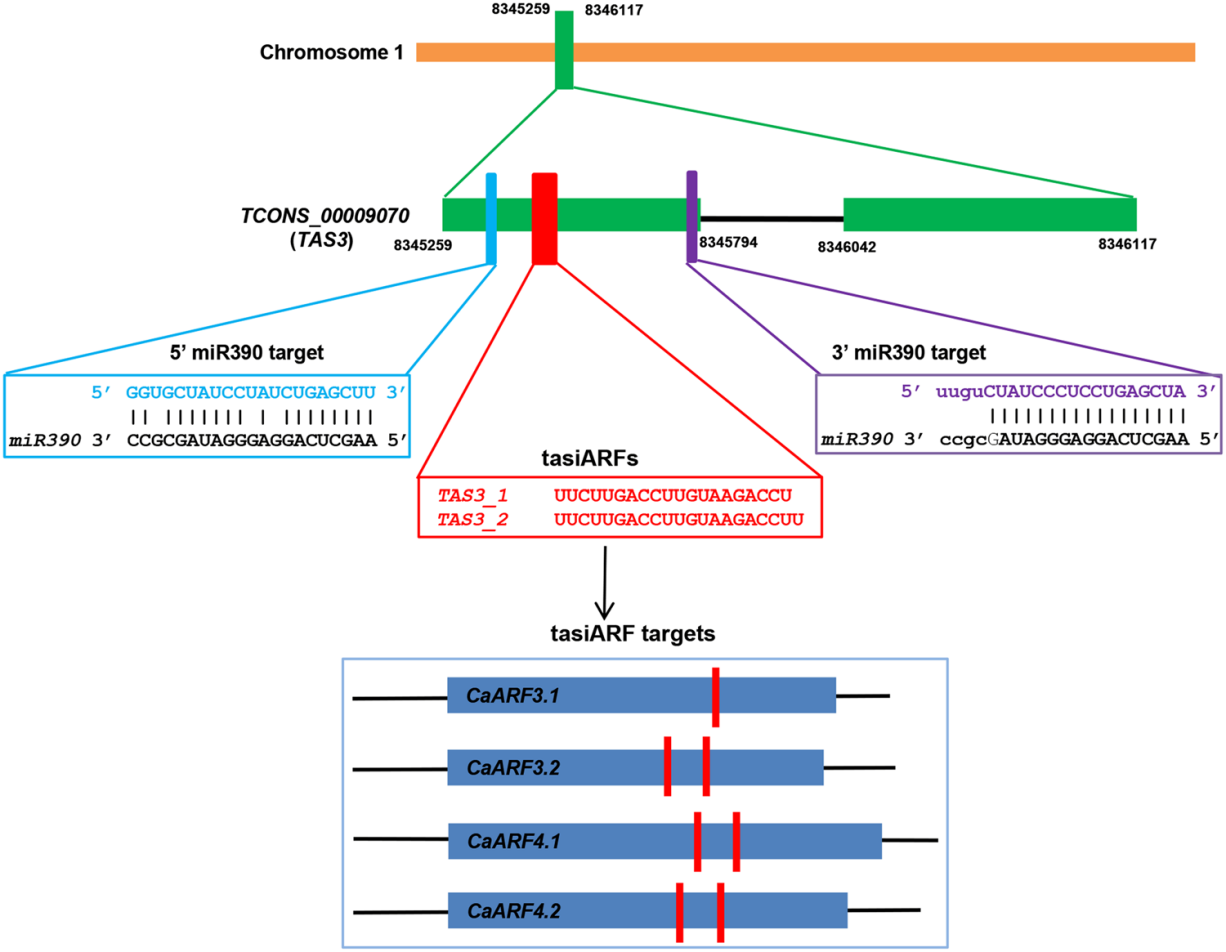
Target sites of tasiRNA in CaARF genes. Diagram showing *TAS3* locus with miRNA390 target sites (blue and purple boxes) and tasiARF biogenesis site (red box). tasiARF targets on CaARFs are shown by red lines.

Further, we investigated *miR390*-*TAS3*-*ARF* pathway in chickpea. In plants, miR390 slices *TRANS-ACTING SIRNA 3* (*TAS3*) transcripts to produce tasiRNAs that regulate *ARF* genes. In Arabidopsis, TAS3-derived tasiARF targets are, *AtARF2*, *AtARF3* and *AtARF4* ^63^. This *miR390*-*TAS*3-*ARF* pathway performs crucial function in regulation of plant growth and development, including lateral root growth, leaf morphogenesis, developmental timing and patterning^64–68^. For the identification of *TAS3* locus in chickpea, we annotated genomic loci harbouring *miR390* target sites and tasiRNAs that target *ARF* gene(s). We identified one genomic locus (*TCONS_00009070*), with two target sites of *miR390* and two tasiRNAs in phased manner targeting *CaARFs* (*CaARF3*.*1*, *3*.*2*, *4*.*1* and *4*.*2*) (Fig. 8). Interestingly, TAS3-derived tasiRNA targets in chickpea, *CaARF3*.*1*, *3*.*2*, *4*.*1* and *4*.*2*, belonged to same phylogenetic clade as reported for Arabidopsis (*AtARF3* and *4*) too^61^. Taken together, these analyses suggested a role of small RNA pathways in regulation of CaARFs and biogenesis of tasiRNAs, known for regulation of their ARF targets.

In conclusion, we identified 28 chickpea ARF genes and established the classification and evolutionary relationship among these genes via phylogenetic tree, gene structure and conserved protein motif analyses. Expression analyses highlighted the involvement of CaARF genes in flower development and response to abiotic stress. Furthermore, co-expression network analysis indicated ARF-mediated regulation of Aux/IAA and GH3 genes and suggested their role in developmental processes. We revealed regulation of CaARF genes via identifying targets of miRNAs and tasiRNAs. The data presented here will provide solid foundation for future studies on the functional characterization of ARF genes and ARF-mediated signal transduction pathways in chickpea.

## Methods

### Identification of putative *ARF* genes in chickpea genome

Kabuli chickpea (*Cicer arietinum* L.) genome annotation was downloaded from Legume Information system (LIS; http://cicar.comparative-legumes.org/)^69^. For identification of ARF gene family members, BLASTP and HMM profile searches were employed. For BLASTP, 23 Arabidopsis ARF protein sequences were taken as query and searched in the chickpea proteome with e-value cut-off ≤1e^−5^. For HMM search, HMM profile of ARF domain (PF00025) was downloaded from Pfam database (http://pfam.sanger.ac.uk/search). The gene ids obtained from these two searches were combined to make a non-redundant list and their protein sequences were analyzed in Pfam and SMART databases for the presence of ARF domain. Properties of chickpea ARF proteins were analyzed using ExPASy server (http://web.expasy.org/compute_pi/).

### Prediction of gene structure and motifs, and phylogenetic analysis

Exon/intron organization of the ARF genes was determined using Gene Structure Display Server (http://gsds.cbi.pku.edu.cn/) using GFF file. The motifs of chickpea ARF protein sequences were analyzed via MEME programme^70^ (http://meme-suite.org/tools/meme) with motif length 6–100 and number of sites 2–120 with maximum number of motifs set to 15. For phylogenetic analysis, protein sequence of ARFs, from chickpea and Arabidopsis were aligned using MUSCLE multiple sequence alignment tool with default settings. Unrooted phylogenetic tree construction was done by Neighbour-Joining (NJ) method using MEGA (v7) software with following parameters: JTT model, pair-wise gap deletion and 100 bootstrap.

### Localization of genes on chromosomes and gene duplication analysis

Information about location of chromosomes was obtained from the genome annotation GFF file. Tandem and segmental duplication of chickpea *ARF* genes were analyzed using MCScanX software. The *Ka* (nonsynonymous substitution rate) and *Ks* (synonymous substitution rate) were calculated using perl script add_ka_and_ks_to_synteny.pl from MCSanX. The *Ks* value was used to calculate the tentative date of duplication event (*T* = *Ks*/2*λ*) assuming clock-like substitution of 6.05 × 10^−9^ substitutions/synonymous site/year for chickpea. Mapping of genes and segmentally duplicated regions on the chickpea chromosomes was done using Circos tool^71^.

### RNA-seq data for gene expression analysis of chickpea ARFs and RT-qPCR

To study gene expression profiles of chickpea ARFs, we used RNA-seq data from previous studies^36, 37^. RNA-seq data from 17 tissues/organs, including germinating seedling (GS), root (R), shoot (S), stem (ST), mature leaf (ML), young leaf (YL), shoot apical meristem (SAM), stages of flower bud (FB1-4; where FB1, FB2, FB3 and FB4 represent 4 mm, 6 mm, 8 mm and 8–10 mm size flower buds, respectively), stages of flower (FL1-5; where FL1 = young flower with closed petals, FL2 = flower with partially opened petals, FL3 = mature flower, FL4 = mature flower with opened and faded petal and FL5 = drooped flower with senescing petals) and young pod (YP), were analyzed as described earlier^72^. Expression analysis under abiotic stress was performed as described^72^, where root and shoot tissues were analyzed under control and stress (desiccation, salt and cold) conditions, using RNA-seq data as described^44^.

Various tissue samples used in RNA-seq analysis were collected as described^36, 37^. Total RNA was extracted from tissues using Tri Reagent (Sigma-Aldrich) followed by synthesis of cDNA using 3 µg of total RNA as described^72^. Primer pairs used in qPCR were designed using Primer Express software according to manufacturer’s guidelines (Applied Biosystem, USA) and are listed in Table S7. qPCR reactions for each tissue sample were performed in at least two biological replicates and three technical replicates for each biological replicate employing ABI 7500 system (Applied Biosystems) as described earlier^73^. *Elongation factor-1 alpha* (*EF-1α*) was used as endogenous control for normalization of transcript levels across the tissue samples.

### Co-expression network construction

Weighted gene correlation network analysis (WGCNA)^46^ method was employed for constructing a co-expression network among *CaARFs*, *CaIAAs* ^72^ and *CaGH3s*. GH3 genes in the kabuli chickpea genome (CaGH3s) were identified as described previously^74^ (Table S8). WGCNA presents a systematic method for examining possible related genes acting in a common pathway, using gene expression data^46^. Firstly, adjacency matrix between CaARF, CaIAA and CaGH3 genes was calculated using expression data on the basis of Pearson correlation coefficient^46^. The following formula depicts how adjacent values between the two genes can be expressed^46^: a_ij_ = |cor(*x*_*i*_, *x*_*j*_))|^β^, where a_*ij*_ represents the adjacency value between gene *i* and gene *j*; cor(*x*_*i*_, *x*_*j*_) is the Pearson correlation coefficient between gene *i* and *j*; β represents the weight value. TOM similarity algorithm was used to convert the adjacency matrix to a topological overlap (TO) matrix, which signifies gene correlation in a network. Hierarchical clustering was performed using dissimilarity matrix, the inverse matrix of TO value (1–TO), for representing genetic link network. The dynamic tree-cut algorithm was used for cutting the hierarchical clustered tree into branches corresponding to diverse modules^46^. Graphical representation of the network was done using VisANT^75^.

### Promoter sequence analysis

Promoter sequences (1000 bp upstream from the start codon) of all ARF genes from chickpea were subjected to search in PlantPAN 2.0 database^42^ (http://plantpan2.itps.ncku.edu.tw/) for identification of *cis*-regulatory elements.

## Electronic supplementary material

Supplementary Informtion
