# Supplementary material for: Genome-wide identification and co-expression network analysis provide insights into the roles of auxin response factor gene family in chickpea
Source: Sci Rep. 2017 Sep 7;7:10895. doi: 10.1038/s41598-017-11327-5 (PMC5589731; doi:10.1038/s41598-017-11327-5)
Supplement: Supplementary file 1 — Supplementary Informtion [file 41598_2017_11327_MOESM1_ESM.pdf]

**Supplemental Information**

*Correspondence and requests for materials should be addressed to

M.J. (mjain@jnu.ac.in)

# Genome-wide identification and co-expression network analysis provide insights into the roles of auxin response factor gene family in chickpea

Vikash K. Singh[1], Mohan Singh Rajkumar[1], Rohini Garg[2], Mukesh Jain[1, 3]*

[1]National Institute of Plant Genome Research (NIPGR), New Delhi, India

[2]School of Natural Sciences, Department of Life Sciences, Shiv Nadar University (SNU), Uttar Pradesh, India

[3]School of Computational and Integrative Sciences (SC&IS), Jawaharlal Nehru University (JNU), New Delhi, India

**Table S1** List of ARF genes identified in chickpea and their sequence characteristics.

| Gene name | Gene identifier | Chr./Scaffolds | Strand | Start | Stop | CDS length (bp) | Protein | | |
|---|---|---|---|---|---|---|---|---|---|
| | | | | | | | Length (aa) | pI | Mol. wt. (kDa) |
| *CaARF1* | *Ca_00010* | Ca1 | + | 154942 | 160324 | 2025 | 674 | 5.66 | 75.33 |
| *CaARF2* | *Ca_05681* | Ca6 | - | 6142286 | 6146054 | 2454 | 817 | 6.24 | 91.5 |
| *CaARF3.1* | *Ca_06352* | Ca6 | - | 17366001 | 17370933 | 2172 | 723 | 6.32 | 80.1 |
| *CaARF3.2* | *Ca_00347* | Ca1 | - | 2832858 | 2837335 | 2100 | 699 | 6.13 | 76.25 |
| *CaARF4.1* | *Ca_14590* | Ca6 | - | 27668057 | 27673700 | 2424 | 807 | 6.53 | 89.81 |
| *CaARF4.2* | *Ca_02516* | Ca1 | + | 12333559 | 12337552 | 2229 | 742 | 8.01 | 83.16 |
| *CaARF5.1* | *Ca_10748* | Ca4 | - | 48381171 | 48385899 | 2754 | 917 | 5.55 | 102.51 |
| *CaARF5.2* | *Ca_10794* | Ca4 | + | 47957191 | 47959645 | 840 | 279 | 8.24 | 31.25 |
| *CaARF5.3* | *Ca_10795* | Ca4 | + | 47936906 | 47950021 | 1272 | 423 | 6.35 | 47.1 |
| *CaARF5.4* | *Ca_04827* | Ca5 | - | 31213397 | 31217778 | 2064 | 687 | 5.78 | 75.71 |
| *CaARF5.5* | *Ca_10790* | Ca4 | - | 47971792 | 47988044 | 2286 | 761 | 6.22 | 84.41 |
| *CaARF6.1* | *Ca_00467* | Ca1 | - | 3851742 | 3857196 | 2748 | 915 | 6.22 | 101.58 |
| *CaARF6.2* | *Ca_05025* | Ca6 | + | 12433367 | 12438750 | 2754 | 917 | 6.17 | 102.75 |
| *CaARF7.1* | *Ca_03128* | Ca7 | - | 3354150 | 3360236 | 2973 | 990 | 6.33 | 110.78 |
| *CaARF7.2* | *Ca_19289* | Ca1 | - | 24068740 | 24075356 | 2493 | 830 | 5.7 | 92.16 |
| *CaARF8.1* | *Ca_15694* | Ca2 | - | 28016569 | 28023962 | 2502 | 833 | 5.89 | 92.56 |
| *CaARF8.2* | *Ca_08872* | Ca5 | - | 27748636 | 27757740 | 2694 | 897 | 5.67 | 99.65 |
| *CaARF9.1* | *Ca_21948* | Ca6 | + | 41935868 | 41941052 | 2064 | 687 | 6.39 | 76.19 |
| *CaARF9.2* | *Ca_23296* | Ca3 | + | 14022456 | 14026905 | 1383 | 460 | 5.9 | 51.88 |
| *CaARF9.3* | *Ca_17636* | Ca7 | - | 35086618 | 35091249 | 2040 | 679 | 6.38 | 76.1 |
| *CaARF10.1* | *Ca_26121* | Ca6 | - | 28197079 | 28200595 | 2121 | 706 | 7.03 | 78.58 |
| *CaARF10.2* | *Ca_02541* | Ca1 | + | 12146278 | 12149182 | 2160 | 719 | 8.56 | 79.44 |
| *CaARF16.1* | *Ca_17136* | Ca4 | - | 20117127 | 20119723 | 2079 | 692 | 7.05 | 77.44 |
| *CaARF16.2* | *Ca_08488* | Ca4 | + | 10173514 | 10176172 | 1836 | 611 | 7.58 | 67.93 |
| *CaARF16.3* | *Ca_17624* | Ca7 | + | 34900006 | 34901988 | 1716 | 571 | 7.28 | 64.73 |
| *CaARF17.1* | *Ca_11175* | scaffold1301_1 | - | 135142 | 136967 | 1350 | 449 | 6.18 | 49.58 |
| *CaARF17.2* | *Ca_14329* | Ca2 | + | 29095100 | 29096692 | 1515 | 504 | 6.63 | 55.43 |
| *CaARF19* | *Ca_05876* | Ca6 | + | 4214744 | 4221311 | 3270 | 1089 | 6.18 | 120.33 |

**Table S2** (a) List of motifs identified in each CaARF (b) Sequence logo of different motifs identified through MEME.

**(a)**

| Gene name | E-value | Motifs |
|---|---|---|
| CaARF1 | 6.00E-246 | 3,5,1,11,9,7,6,12,4,10,8 |
| CaARF2 | 4.50E-271 | 3,5,1,11,2,9,7,6,12,4,10,8 |
| CaARF3.1 | 2.70E-178 | 3,5,1,11,2,9,7,13,6,12 |
| CaARF3.2 | 4.20E-170 | 3,5,1,9,7,13,6,12 |
| CaARF4.1 | 3.20E-265 | 3,5,1,11,2,9,7,13,6,12,4,10,8 |
| CaARF4.2 | 1.10E-177 | 3,5,11,2,9,7,13,6,12,4,10 |
| CaARF5.1 | 0 | 3,5,1,11,2,9,7,13,6,12,4,10,8 |
| CaARF5.2 | 1.10E-187 | 3,5,14,2,7,13,6 |
| CaARF5.3 | 3.20E-195 | 3,14,9,7,6,12,4 |
| CaARF5.4 | 7.20E-51 | 11,2,9,7 |
| CaARF5.5 | 7.40E-141 | 5,9,7,13,6,12 |
| CaARF6.1 | 0 | 3,5,1,11,2,9,7,13,6,12,4,14,10,8 |
| CaARF6.2 | 0 | 3,5,1,11,2,9,7,13,6,12,4,14,10,8 |
| CaARF7.1 | 0 | 3,5,1,11,2,9,7,13,6,12,4,10,8 |
| CaARF7.2 | 0 | 3,5,1,11,2,9,7,13,6,12,4,10,8 |
| CaARF8.1 | 0 | 3,5,1,11,2,9,7,13,6,12,4,14,10,8 |
| CaARF8.2 | 0 | 3,5,1,11,2,9,7,13,6,12,4,14,10,8 |
| CaARF9.1 | 2.50E-261 | 3,5,1,11,2,9,7,6,12,4,10,8 |
| CaARF9.2 | 9.90E-264 | 3,5,1,11,2,9,7,6,12,4,10,8 |
| CaARF9.3 | 2.50E-267 | 3,5,1,11,2,9,7,6,12,4,10,8 |
| CaARF10.1 | 3.40E-214 | 3,5,1,11,2,9,7,6,4,10,12 |
| CaARF10.2 | 3.10E-217 | 3,5,1,11,2,9,7,6,12,4,10 |
| CaARF16.1 | 1.50E-208 | 3,5,1,11,2,9,7,6,12,4,10 |
| CaARF16.2 | 3.80E-150 | 3,5,11,2,9,7,6,12,4 |
| CaARF16.3 | 5.20E-167 | 3,5,1,11,2,9,7,6,12,4 |
| CaARF17.1 | 4.70E-73 | 11,2,9,7,6,4 |
| CaARF17.2 | 2.40E-118 | 3,5,1,11,2,9,7,4 |
| CaARF19 | 4.10E-265 | 3,5,1,11,9,7,13,6,12,4,10,8 |

**(b)**

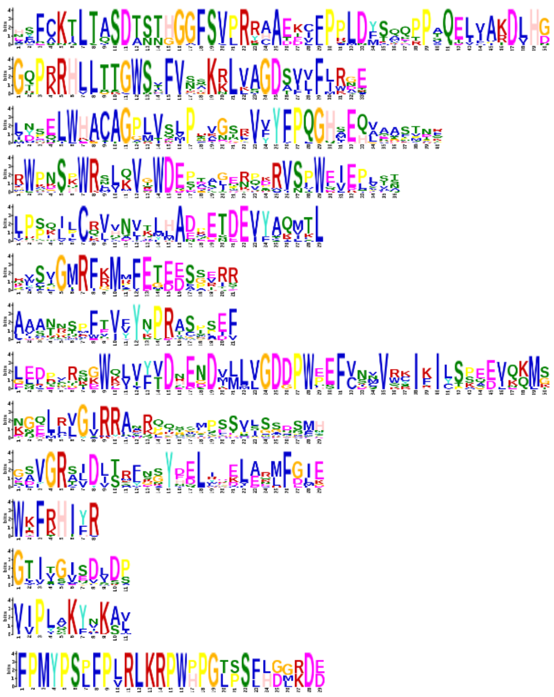

| | Logo | E-value | Sites | Width |
|---|---|---|---|---|
| 1. | | 4.9e-676 | 21 | 50 |
| 2. | | 1.8e-491 | 23 | 33 |
| 3. | | 7.8e-535 | 25 | 40 |
| 4. | | 4.5e-394 | 23 | 36 |
| 5. | | 1.4e-389 | 26 | 29 |
| 6. | | 3.8e-275 | 26 | 21 |
| 7. | | 7.6e-264 | 28 | 21 |
| 8. | | 4.0e-343 | 14 | 50 |
| 9. | | 3.5e-219 | 27 | 29 |
| 10. | | 1.2e-204 | 18 | 29 |
| 11. | | 4.4e-133 | 24 | 8 |
| 12. | | 3.2e-075 | 24 | 11 |
| 13. | | 2.0e-051 | 15 | 11 |
| 14. | | 1.3e-034 | 4 | 29 |

**Table S3** Segmentally duplicated CaARF gene pairs.

| Gene name | Gene name | Ka | Ks | Mya | Ka/Ks |
|---|---|---|---|---|---|
| *CaARF3.1* | *CaARF3.2* | 0.2 | 0.56 | 46.66 | 0.35 |
| *CaARF4.1* | *CaARF4.2* | 0.19 | 0.57 | 47.34 | 0.33 |
| *CaARF7.1* | *CaARF7.2* | 0.1 | 0.66 | 54.49 | 0.15 |
| *CaARF8.1* | *CaARF8.2* | 0.08 | 0.57 | 46.72 | 0.14 |
| *CaARF10.1* | *CaARF10.2* | 0.18 | 0.66 | 54.47 | 0.28 |
| *CaARF10.1* | *CaARF16.1* | 0.3 | 1.36 | 112.05 | 0.22 |
| *CaARF10.2* | *CaARF16.1* | 0.33 | 1.42 | 117.72 | 0.24 |
| *CaARF10.2* | *CaARF16.2* | 0.38 | 0.92 | 76.19 | 0.41 |

**Table S4** CaARFs genes with their expression values (FPKM) in various tissues types.

| Gene name | GS | root | shoot | stem | ML | YL | SAM | FB1 | FB2 | FB3 | FB4 | FL1 | FL2 | FL3 | FL4 | FL5 | YP |
|---|---|---|---|---|---|---|---|---|---|---|---|---|---|---|---|---|---|
| *CaARF1* | 35.39 | 58.65 | 34.86 | 25.77 | 29.57 | 21.83 | 44.76 | 25.31 | 23.32 | 19.99 | 19.82 | 17.31 | 21.21 | 24.97 | 22.19 | 22.49 | 38.53 |
| *CaARF2* | 58.79 | 135.74 | 71.14 | 45.91 | 76.05 | 46.1 | 103.96 | 87.47 | 66.84 | 51.24 | 40.31 | 29.6 | 34.31 | 36.9 | 36.24 | 42.19 | 93.12 |
| *CaARF3.1* | 20.32 | 4.96 | 12.24 | 32.82 | 4.78 | 8.57 | 10.77 | 21.42 | 16.02 | 13.17 | 16.95 | 17.14 | 12.71 | 9.33 | 11.33 | 9.78 | 49.03 |
| *CaARF3.2* | 21.87 | 9.33 | 11.91 | 38.76 | 3.21 | 15.41 | 41.95 | 25.79 | 17.76 | 19.16 | 21.08 | 13.85 | 12.07 | 11.1 | 10.42 | 12.68 | 17.24 |
| *CaARF4.1* | 13.71 | 12.88 | 17.25 | 18.04 | 6.67 | 18.66 | 12.98 | 19.24 | 14.86 | 11.44 | 11.06 | 10.38 | 10.13 | 12.82 | 13.94 | 14.67 | 17.17 |
| *CaARF4.2* | 5.79 | 9.71 | 7.95 | 13.1 | 0.34 | 1.56 | 5.05 | 4.27 | 3.78 | 2.97 | 3.5 | 3.02 | 3.41 | 3.53 | 2.01 | 3.92 | 5.2 |
| *CaARF5.1* | 18.56 | 6.58 | 8.13 | 4.09 | 0.05 | 9.56 | 31.29 | 23.6 | 12.57 | 9.12 | 6.86 | 7.18 | 8.22 | 7.76 | 10.62 | 13.69 | 28.64 |
| *CaARF5.3* | 0 | 0 | 0 | 0 | 0 | 0 | 0 | 0.02 | 0 | 0 | 0 | 0 | 0 | 0 | 0 | 0 | 0 |
| *CaARF5.4* | 0.33 | 0.34 | 0.63 | 0.45 | 0.56 | 0.92 | 0.18 | 1.19 | 0.79 | 0.79 | 0.57 | 0.53 | 0.36 | 0.31 | 0.14 | 0.2 | 0.4 |
| *CaARF5.5* | 0.07 | 0.02 | 0 | 0.02 | 0 | 0 | 0.06 | 0.48 | 1.22 | 1.62 | 0.79 | 0.15 | 0.16 | 0.05 | 0.02 | 0.03 | 0.04 |
| *CaARF6.1* | 31.67 | 29.78 | 48.17 | 63.93 | 22.38 | 47.17 | 30.01 | 41.43 | 34.65 | 35.56 | 38.51 | 33.84 | 37.88 | 34.78 | 25.04 | 30.46 | 57.71 |
| *CaARF6.2* | 27.16 | 20.92 | 37.46 | 69.4 | 13.87 | 40.57 | 34.09 | 46.63 | 37.74 | 36.41 | 42.93 | 66.08 | 65.92 | 48.69 | 27.33 | 34.47 | 32.4 |
| *CaARF7.1* | 19.92 | 19.36 | 13.6 | 13.05 | 25.04 | 2.69 | 4.12 | 5.22 | 7.37 | 6.01 | 9.23 | 27.14 | 28.59 | 25.02 | 20.62 | 16.79 | 13.52 |
| *CaARF7.2* | 29.49 | 61.96 | 50.5 | 42.83 | 16.77 | 35.51 | 27.06 | 10.11 | 8.91 | 8.8 | 10.14 | 12.84 | 13.23 | 21.48 | 16.92 | 23.2 | 31.78 |
| *CaARF8.1* | 14.84 | 5.81 | 11.26 | 41.98 | 1.09 | 20.76 | 27.71 | 38.46 | 29.03 | 25.91 | 23.04 | 14.97 | 11.95 | 11.17 | 12.27 | 13.54 | 55.47 |
| *CaARF8.2* | 1427.9 | 737.52 | 453.26 | 897.29 | 718.27 | 327.48 | 333.27 | 539.8 | 1311.97 | 1246.96 | 1062.46 | 1232.85 | 1478.42 | 1871.21 | 1146.48 | 1500.99 | 1075.13 |
| *CaARF9.1* | 21.35 | 91.22 | 24.55 | 17.67 | 40.03 | 19.5 | 21.37 | 26.69 | 14.8 | 12.98 | 8.13 | 8.18 | 10.03 | 13.22 | 24.84 | 17.68 | 22.15 |
| *CaARF9.2* | 23.19 | 102.98 | 10.53 | 3.9 | 8 | 15.32 | 56.56 | 40.66 | 20.06 | 9.95 | 7.07 | 7.45 | 8.93 | 10.85 | 13.38 | 15.83 | 6.04 |
| *CaARF9.3* | 9.8 | 28.57 | 14.42 | 8.9 | 10.68 | 14.36 | 33.04 | 36.71 | 11.76 | 6.28 | 4.11 | 3.3 | 3.78 | 3.42 | 2.95 | 4.39 | 0.51 |
| *CaARF10.1* | 2.94 | 5.15 | 3.31 | 4.77 | 4.21 | 3.42 | 3.98 | 5.13 | 4.05 | 4.8 | 6.2 | 4.2 | 5.27 | 4.36 | 4.74 | 3.89 | 5.36 |
| *CaARF10.2* | 3.9 | 1.98 | 5.7 | 12.45 | 6.64 | 9.01 | 8.78 | 13.2 | 9.61 | 8.05 | 9.11 | 8.28 | 7.92 | 5.38 | 6.21 | 5.41 | 4.4 |
| *CaARF16.1* | 6.29 | 6.25 | 8.65 | 8.88 | 11.32 | 9.73 | 5.08 | 10.43 | 10.12 | 9.95 | 14.04 | 15.92 | 14.14 | 13.2 | 12.05 | 10.22 | 6.69 |
| *CaARF16.2* | 4.23 | 3.22 | 2.5 | 6.76 | 1.96 | 4.16 | 2.16 | 3.65 | 3.02 | 4.47 | 7.24 | 6.55 | 5.73 | 3.96 | 3.29 | 2.97 | 4.6 |
| *CaARF16.3* | 0.03 | 0 | 0 | 0.26 | 0 | 0.03 | 0.02 | 5.37 | 0.12 | 0.07 | 0.08 | 0.09 | 0.05 | 0.04 | 0.07 | 0 | 0.04 |
| *CaARF17.1* | 3.62 | 7.05 | 5.64 | 6.17 | 5.83 | 5.33 | 6.77 | 10.72 | 4.82 | 3.84 | 4.54 | 3.01 | 2.93 | 4.49 | 8.63 | 6.06 | 6.12 |
| *CaARF17.2* | 0.24 | 0.28 | 0.3 | 0.2 | 0.14 | 0 | 0.08 | 3.39 | 0.73 | 0.24 | 0.21 | 0.14 | 0.11 | 0.19 | 0.11 | 0.11 | 0.14 |
| *CaARF19* | 21.17 | 31.85 | 35.68 | 41.11 | 26.97 | 23.84 | 15.51 | 12.75 | 10.61 | 10.61 | 17.36 | 55.23 | 56.73 | 32.05 | 13.44 | 10.22 | 15.84 |

**Table S5** Motifs identified in CaARF promoter sequences.

| Gene name | Motifs |
|---|---|
| *CaARF1* | ABRELATERD1, GAREAT, WBOXATNPR1 |
| *CaARF2* | ABRELATERD1, B2GMAUX28, C2GMAUX28, CAATBOX1, D3GMAUX28, DRECRTCOREAT, GAREAT, GCCCORE, GT1GMSCAM4, TCA1MOTIF, T/GBOXATPIN2, WBOXATNPR1 |
| *CaARF3.1* | ABRELATERD1, C2GMAUX28, CAATBOX1, D3GMAUX28, GAREAT, GT1GMSCAM4, WBOXATNPR1 |
| *CaARF3.2* | ABRELATERD1, GAREAT, WBOXATNPR1 |
| *CaARF4.1* | ABRELATERD1, B2GMAUX28, C2GMAUX28, CAATBOX1, D1GMAUX28, D3GMAUX28, DRECRTCOREAT, GAREAT, GT1GMSCAM4, TATABOX1, TCA1MOTIF, T/GBOXATPIN2, WBOXATNPR1 |
| *CaARF4.2* | ABRELATERD1, GAREAT, WBOXATNPR1 |
| *CaARF5.1* | ABRELATERD1, B2GMAUX28, C2GMAUX28, CAATBOX1, D1GMAUX28, D3GMAUX28, GAREAT, GT1GMSCAM4, TATABOX1, T/GBOXATPIN2, WBOXATNPR1 |
| *CaARF5.2* | ABREATRD22, ABRELATERD1, GAREAT, T/GBOXATPIN2, WBOXATNPR1 |
| *CaARF5.3* | ABRELATERD1, GAREAT, WBOXATNPR1 |
| *CaARF5.4* | ABRELATERD1, WBOXATNPR1 |
| *CaARF5.5* | ABRELATERD1, B2GMAUX28, C2GMAUX28, CAATBOX1, D3GMAUX28, GAREAT, GT1GMSCAM4, TATABOX1, T/GBOXATPIN2, WBOXATNPR1 |
| *CaARF6.1* | ABRELATERD1, GAREAT, WBOXATNPR1 |
| *CaARF6.2* | ABRELATERD1, B2GMAUX28, C2GMAUX28, CAATBOX1, D1GMAUX28, D3GMAUX28, DRECRTCOREAT, GAREAT, GCCCORE, GT1GMSCAM4, TCA1MOTIF, WBOXATNPR1 |
| *CaARF7.1* | ABRELATERD1, B2GMAUX28, C2GMAUX28, CAATBOX1, D1GMAUX28, D3GMAUX28, GAREAT, GT1GMSCAM4, WBOXATNPR1 |
| *CaARF7.2* | ABRELATERD1, GAREAT, WBOXATNPR1 |
| *CaARF8.1* | ABRELATERD1, GAREAT, WBOXATNPR1 |
| *CaARF8.2* | ABRELATERD1, BOXIIPCCHS, C2GMAUX28, CAATBOX1, D1GMAUX28, D3GMAUX28, DRECRTCOREAT, GAREAT, GT1GMSCAM4, LTRE1HVBLT49, T/GBOXATPIN2, WBOXATNPR1 |
| *CaARF9.1* | ABRELATERD1, B2GMAUX28, C2GMAUX28, CAATBOX1, D1GMAUX28, D3GMAUX28, GAREAT, GT1GMSCAM4, WBOXATNPR1 |
| *CaARF9.2* | ABRELATERD1, GAREAT, T/GBOXATPIN2, WBOXATNPR1 |
| *CaARF10.1* | ABRELATERD1, B2GMAUX28, C2GMAUX28, CAATBOX1, CBFHV, D1GMAUX28, D3GMAUX28, DRECRTCOREAT, GAREAT, GT1GMSCAM4, TATABOX1, TCA1MOTIF, WBOXATNPR1 |
| *CaARF10.2* | ABRELATERD1, GAREAT, WBOXATNPR1 |
| *CaARF16.1* | ABREATCONSENSUS, ABREATRD22, ABRELATERD1, GAREAT, GBOXLERBCS, WBOXATNPR1 |
| *CaARF16.2* | ABRELATERD1, WBOXATNPR1 |
| *CaARF16.3* | ABRELATERD1, B2GMAUX28, BOXIIPCCHS, C2GMAUX28, CAATBOX1, CBFHV, D1GMAUX28, D3GMAUX28, DRECRTCOREAT, GAREAT, GT1GMSCAM4, WBOXATNPR1 |
| *CaARF17.2* | ABRELATERD1, GAREAT, WBOXATNPR1 |
| *CaARF19* | ABRELATERD1, B2GMAUX28, C2GMAUX28, CAATBOX1, CBFHV, D1GMAUX28, D3GMAUX28, GAREAT, GCCCORE, GT1GMSCAM4, WBOXATNPR1 |

**Table S6** Ca*ARFs* genes with their $\log_2$ fold change value with respect to control in different stress condition.

| Gene name | Root-DS | Root-SS | Root-CS | Shoot-DS | Shoot-SS | Shoot-CS |
|---|---|---|---|---|---|---|
| *CaARF4.2* | -0.48 | 0.27 | 0.32 | -0.12 | -0.22 | -0.99 |
| *CaARF7.1* | 0.14 | 0.43 | 0.36 | 2.46 | 0.15 | -0.06 |
| *CaARF9.2* | 1.90 | -0.01 | 1.95 | 0.06 | -0.27 | -0.25 |
| *CaARF16.2* | 2.10 | 0.96 | 2.67 | -2.21 | -0.27 | 0.26 |

DS, dessication stress; SS, salt stress; CS, Cold stress

**Table S7** Primer sequences of *CaARF* genes used for RT-qPCR.

| Gene name | Primer sequence |
|---|---|
| *CaARF4.2* | F-TCTCTCCCTCCGTTGAACATTC<br>R-AGACCCGTCCGCAGTTTCTT |
| *CaARF5.3* | F-TGGTACGCGCAGATATTTGG<br>R-CAGGCCACATTAGAGGATCCA |
| *CaARF5.5* | F-CGGTCCTTCGCAAGTAAAGAGT<br>R-CCGGGTCTGGATAGGTCAAG |
| *CaARF7.1* | F-CAACGAATGCGGACATATACTAAGG<br>R-TGACGTCAATGCATCTTCCAA |
| *CaARF9.2* | F-AGCTGCATGCCTTTATCTGTGA<br>R-TGCAGTTGCAAGGACTCCAA |
| *CaARF10.1* | F-TTTTGTGTTAAGGCTTCAGCTGTT<br>R-CATCTTGAACCTCATCCCAGAAC |
| *CaARF16.2* | F-GCAGACCCTGAAACAGATGAAGT<br>R-TCTGAATCTAATTCGTGGTGTTCTCT |
| *CaARF16.3* | F-CACTTTTTTTGAGCCTATTGATCCA<br>R-CATGTAACCTGAAGAGCTCTCCAA |
| *CaARF17.2* | F-GGTGGATGCTAAGGTGGTTGA<br>R-CAAAGCTTCACTCTCATTCCACAA |
| *EF1α* | F-TCCACCACTTGGTCGTTTTG<br>R-CTTAATGACACCGACAGCAACAG |

**Table S8** List of GH3 genes identified in kabuli chickpea.

| Gene name | Gene identifier | Chr./Scaffolds | Strand | Start | Stop |
|---|---|---|---|---|---|
| *CaGH3-1* | *Ca_14096* | Ca1 | - | 13341168 | 13344529 |
| *CaGH3-2* | *Ca_14097* | Ca1 | - | 13346034 | 13347264 |
| *CaGH3-3* | *Ca_06937* | Ca1 | - | 16184624 | 16187533 |
| *CaGH3-4* | *Ca_15967* | Ca2 | - | 18653600 | 18655967 |
| *CaGH3-5* | *Ca_07346* | Ca3 | - | 30310314 | 30312308 |
| *CaGH3-6* | *Ca_01301* | Ca3 | + | 39372005 | 39375435 |
| *CaGH3-7* | *Ca_03444* | Ca4 | + | 7924564 | 7927594 |
| *CaGH3-8* | *Ca_23631* | Ca7 | + | 25761776 | 25764566 |
| *CaGH3-9* | *Ca_02190* | Ca8 | - | 3842646 | 3844855 |
| *CaGH3-10* | *Ca_21252* | scaffold1981 | - | 64343 | 66453 |
| *CaGH3-11* | *Ca_21255* | scaffold1981 | - | 103242 | 105804 |
| *CaGH3-12* | *Ca_21256* | scaffold1981 | - | 137176 | 140084 |
